# Vitamin D Supplementation to Prevent COVID-19 Infections and Deaths—Accumulating Evidence from Epidemiological and Intervention Studies Calls for Immediate Action

**DOI:** 10.3390/nu13020411

**Published:** 2021-01-28

**Authors:** Hermann Brenner

**Affiliations:** 1 German Cancer Research Center, Division of Clinical Epidemiology and Aging Research, 69120 Heidelberg, Germany; h.brenner@dkfz.de; Tel.: +49-6221-42-1300; 2 Network Aging Research, Heidelberg University, 69115 Heidelberg, Germany

**Keywords:** COVID-19, mortality, prevention, supplementation, vitamin D

## Abstract

The COVID-19 pandemic poses an unprecedented threat to human health, health care systems, public life, and economy around the globe. The repertoire of effective therapies for severe courses of the disease has remained limited. A large proportion of the world population suffers from vitamin D insufficiency or deficiency, with prevalence being particularly high among the COVID-19 high-risk populations. Vitamin D supplementation has been suggested as a potential option to prevent COVID-19 infections, severe courses, and deaths from the disease, but is not widely practiced. This article provides an up-to-date summary of recent epidemiological and intervention studies on a possible role of vitamin D supplementation for preventing severe COVID-19 cases and deaths. Despite limitations and remaining uncertainties, accumulating evidence strongly supports widespread vitamin D supplementation, in particular of high-risk populations, as well as high-dose supplementation of those infected. Given the dynamics of the COVID-19 pandemic, the benefit–risk ratio of such supplementation calls for immediate action even before results of ongoing large-scale randomized trials become available.

## 1. Introduction

The COVID-19 pandemic poses an unprecedented threat to human health, health care systems, public life, and economy around the globe. More than 1.7 million people died from SARS-CoV-2 infections by December 2020 [1], with daily numbers of deaths rapidly re-increasing in fall of 2020 in many countries on the northern hemisphere. Options to effectively treat severe cases remain very limited, while intensive care needs stretch or overstretch available capacities in many countries. There are hopes to cope with the pandemic with newly developed vaccines, but it will take many more months before vaccines with proven efficacy and safety will be globally available.

Readily available measures to limit the toll of the pandemic are thus of paramount importance. Based on a comprehensive review of the evidence on the role of vitamin D in preventing the toll of respiratory infections from the pre-COVID-19 era, Grant et al. recommended vitamin D supplementation for both preventing and treating COVID-19 infections already at the onset of the pandemic [2]. However, such supplementation has not been widely implemented. This article provides an up-to-date summary of epidemiological and intervention studies conducted during the COVID-19 pandemic on a possible role of vitamin D supplementation for preventing COVID-19 cases and deaths, by either preventing COVID-19 infections, preventing severe course of the disease and deaths among those infected, or both. Finally, an outlook will be given on ongoing trials, and public health and clinical implications of current evidence are discussed.

## 2. Potential Role of Vitamin D Supplementation for Preventing COVID-19 Infection

In a study of 349,598 participants of the U.K. Biobank participants with known baseline vitamin D levels, 449 confirmed COVID-19 infections occurred from 16 March 2020 to 14 April 2020 [3]. Vitamin D levels were inversely related to the risk of COVID-19 infections in univariate analysis, but this association did not persist after adjustment for covariates in multivariable analyses. By contrast, very strong associations were observed between ethnicity and risk of infection, which were slightly attenuated, but persisted after adjustment for vitamin D status, with adjusted odds ratios of 5.3 and 2.65 for blacks and South Asians versus whites, respectively. However, in this study, vitamin D levels were measured in 2006–2010, i.e., 10–14 years prior to the COVID-19 pandemic, and may have been poor indicators of vitamin D status in 2020. In fact, ethnicity, which is strongly and stably associated with vitamin D status, may have been a much better proxy for vitamin D status in 2020, which may explain the observed patterns.

In a study among 7807 members of a large health maintenance organization in Israel tested for COVID-19 from 1 February to 30 April 2020 and at least one preceding vitamin D measurement, the adjusted odds ratios for low vitamin D levels with COVID-19 positivity and hospitalization due to COVID-19 infection were 1.45 (*p* < 0.001) and 1.95 (*p* = 0.061), respectively [4]. However, in this study, the threshold for low vitamin level was rather high (plasma 25-hydroxy-vitamin D [25(OH)D] < 30 ng/mL), and the low vitamin D group included 85% of the population. As much stronger health effects of insufficient vitamin D levels are commonly seen at levels of vitamin D insufficiency (plasma 25(OH)D < 20 ng/mL) or deficiency (plasma 25(OH)D < 12.5 ng/mL) [5], it would be of utmost interest to complement these analyses by a thorough dose–response analysis.

Such dose–response relationships were evaluated in a cohort of >190,000 patients from the United States in whom results of SARS-CoV-2 results performed mid-March through mid-June 2020 were linked to 25(OH)D results from the preceding 12 months [6]. In this cohort, a clear inverse relationship between circulating 25(OH)D levels and SARS-CoV-2 positivity was observed. The SARS-CoV-2 positivity rate was higher in the 39,190 patients with “deficient” 25(OH)D values (<20 ng/mL) (12.5%, 95% confidence interval (C.I.) 12.2–12.8%) than in the 27,870 patients with “adequate” values (30–34 ng/mL) (8.1%, 95% C.I. 7.8–8.4%) and the 12,321 patients with values ≥55 ng/mL (5.9%, 95% C.I. 5.5–6.4%). Those who had a circulating level of 25(OH)D < 20 ng/mL had a 54% higher positivity rate compared with those who had a blood level of 30–34 ng/mL in multivariable analysis. The risk of SARS-CoV-2 positivity continued to decline until the serum levels reached 55 ng/mL. The relationship persisted across latitudes, races/ethnicities, both sexes, and age ranges.

Inverse associations between vitamin D levels and COVID-19 infection were furthermore consistently reported from a case-control study of 201 hospitalized patients and 201 matched controls from Iran [7] and in a cross-sectional study among 392 healthcare workers from the United Kingdom [8]. Finally, negative correlations between mean levels of vitamin D and COVID-19 infection and mortality rates were also reported from an ecological study including 20 European countries [9].

## 3. Potential Role of Vitamin D Supplementation for Preventing Severe Course of Disease and Death from COVID-19 Infection

A recent clinic-based cohort study among 185 patients diagnosed with and treated for COVID-19 at a University Hospital in Germany showed more than 80% lower risk of invasive mechanical ventilation or death (primary endpoint) and more than 90% lower mortality among patients with sufficient vitamin D levels compared with patients with vitamin D deficiency even after multivariable adjustment for age, gender, and comorbidities [10], suggesting that close to 90% of deaths in this cohort were statistically associated with vitamin D insufficiency [11]. Increased mortality was likewise seen for those with vitamin D levels below the median in a cohort of 30 patients admitted to an intensive care unit in Greece (28-day mortality 5/15 versus 0/15, *p* = 0.01) [12].

In a recent “quasi-experimental” study from France [13], risks of severe course of the disease and of dying within 14 days of admission to a geriatric hospital unit were more than 90% lower among patients who were regularly supplemented with vitamin D over the preceding year (*n* = 29) compared with patients with no vitamin D supplementation (*n* = 32). Despite the limited numbers of cases, strong associations were statistically significant and persisted after multivariable adjustment for confounders. Intermediate and statistically non-significant results were seen for the small group (*n* = 16) of patients who received vitamin D supplementation after COVID-19 diagnosis. In another quasi-experimental study from the same group [14], 82.5% of 57 nursing home residents who received bolus vitamin D3 supplementation either in the week following the suspicion or diagnosis of COVID-19 or during the previous month survived during a mean follow-up of 36 days compared with only 4 out of 9 residents (44%) without such therapy. Despite the small case numbers, the association was highly statistically significant, with an adjusted hazard ratio (95% CI) of 0.11 (0.03–0.48). Vitamin D supplementation was likewise associated with significantly reduced mortality during the COVID-19 pandemic in a cohort of 157 residents of an Italian nursing home [15]. The so far largest “quasi-experimental study” was most recently reported from United Kingdom [16]. A total of 986 participants admitted with COVID-19 to three hospitals were studied, of whom 151 (16.0%) received booster therapy with vitamin D (in its “parent” form, cholecalciferol; approximately 280,000 IU in a time period of up to 7 weeks). In the primary cohort of 444 patients from one hospital, cholecalciferol booster therapy was associated with strongly reduced risk of COVID-19 mortality, with an odds ratio (95% CI) of 0.13 (0.05–0.35, *p* < 0.001) after adjustment for multiple potential confounders. This finding was replicated in a validation cohort of 541 patients from two other hospitals (odds ratio 0.38, 95% CI 0.17–0.84, *p* = 0.018).

However, despite major efforts to control for confounding, such observational or quasi experimental studies remain prone to residual confounding by uncontrolled or imperfectly measured covariates. It is well established that serum 25OHD is a negative acute phase reactant, and associations may in part reflect reverse causality. Such factors could lead to overestimation of the beneficial effects of having adequate vitamin D levels or of vitamin D supplementation. In the “quasi-experimental studies”, confounding by indication, i.e., selective supplementation of vitamin D among those with lowest baseline vitamin D status or in highest need of supplementation, could also lead to underestimation of supplementation effects. On the other hand, if supplementation initiated after the start of mortality follow-up (e.g., several days after diagnosis or hospitalization) is considered as intervention, “immortal time bias” may lead to overestimation of beneficial intervention effects unless appropriate precautions are taken in the analysis, as those with a delayed start of the intervention would necessarily have survived (had been “immortal”) up to such initiation [17]. The final answer as to a causal role of vitamin D supplementation will thus have to come from randomized controlled trials.

A first pilot study of such a randomized trial has been reported from Cordoba, Spain, in which 76 consecutive patients hospitalized with COVID-19, a clinical picture of acute respiratory infection, confirmed by a radiographic pattern of viral pneumonia and by a positive SARS-CoV-2 PCR with CURB65 severity scale (recommending hospital admission in case of total score >1), were enrolled [18]. All hospitalized patients received as best available therapy the same standard care (per hospital protocol) including a combination of hydroxychloroquine and azithromycin. Eligible patients were randomly allocated at a 2:1 ratio on the day of admission to take oral calcifediol (25-hydroxyvitamin D 3, 0.532 mg = 21.280 IU), or not. Patients in the calcifediol treatment group continued with oral calcifediol (0.266 mg = 10.640 IU) on days 3 and 7, and then weekly until discharge or intensive care unit (ICU) admission. Outcomes of effectiveness included the rate of ICU admission and deaths. Only 1 of 50 patients treated with calcifediol (2%), but 13 of 26 untreated patients (50%), required ICU admission, resulting in a multivariate adjusted odds ratio (95% CI) of ICU admission of 0.03 (0.003–0.25). Despite such adjustment, concerns have been expressed with respect to imperfect blinding, as well as uneven distribution of and imperfect control for potential confounders. However, a comprehensive mathematical reanalysis by an independent group concluded that the randomization, large effect size, and high statistical significance address many of these concerns [19]. In particular, it showed that random assignment of patients to treatment and control groups was highly unlikely to distribute comorbidities or other prognostic indicators sufficiently unevenly to account for the large effect size, and that imperfect blinding would need to have had an implausibly large effect to account for the reported results. The authors concluded that the trial provided sufficient evidence to warrant immediate, well-designed pivotal clinical trials of early calcifediol administration in a broader cohort of inpatients and outpatients with COVID-19.

In a more recently published randomized placebo-controlled trial from India [20], high-dose oral cholecalciferol supplementation (60,000 IU per day for at least 7 days in asymptomatic or mildly symptomatic SARS-CoV-2 RNA positive vitamin D deficient (25(OH)D < 20 ng/mL) individuals very effectively overcame vitamin D deficiency (*p* < 0.001), enhanced SARS-CoV-2 viral clearance (*p* = 0.018), and decreased fibrinogen levels (*p* = 0.007). No significant differences were seen for other inflammatory markers. In a multicenter, double-blind, randomized, placebo-controlled trial conducted in two centers (a quaternary hospital and a field hospital) in Sao Paulo, Brazil, involving 240 hospitalized patients with severe COVID-19 (116 with vitamin D deficiency), a single dose of 200,000 IU of vitamin D3 supplementation was likewise safe and effective in increasing 25-hydroxyvitamin D levels, but did not significantly reduce hospital length of stay or any other clinically-relevant outcomes compared with placebo [21]. It has been suggested that oral supplementation with vitamin D3 (a slower-acting treatment than oral supplementation with calcifediol) of this mostly obese population of patients (mean body mass index (BMI) 31.6 kg/m^2^) may have been provided too late to significantly affect clinically relevant outcomes (randomization occurred on average 10 days after onset of symptoms, with 90% of patients requiring supplemental oxygen at baseline) [19].

## 4. Ongoing Trials

Timely conduction, completion, and publication of further well-designed studies including, but not restricted to large-scale randomized clinical trials is paramount for more fully exploring and defining the role of vitamin D supplementation in preventing occurrence and severe course of COVID-19 infections [22]. Several large-scale trials are currently under way, with the main results expected at some time in 2021. Key characteristics of some of the major trials are outlined below.

CORONAVIT, an open-label, phase 3, randomised clinical trial conducted in the United Kingdom, investigates whether implementation of a test-and-treat approach to correction of sub-optimal vitamin D status results in reduced risk and/or severity of COVID-19 and other acute respiratory infections [23]. The trial started on 27 October 2020 and is designed to recruit 6200 U.K. residents ≥16 years. Participants in the intervention group with 25(OH)D level < 30 ng/mL are offered a daily dose of 800 IU or 3200 IU cholecalciferol, while the control group receives standard of care (national recommendation of 400 IU/day vitamin D). The primary endpoint is the proportion of participants experiencing at least one doctor-diagnosed or laboratory-confirmed acute respiratory infection of any cause over 6 months. The secondary endpoints include multiple COVID-19-specific endpoints, such as proportions of participants developing antigen test-positive COVID-19, seroconverting to SARS-CoV-2, requiring hospitalization due to COVID-19, hospitalised for COVID-19 requiring ventilatory support, and dying of COVID-19, along with other or more generic endpoints, such as proportions of participants who experience influenza requiring hospitalization, dying of influenza, dying of any acute respiratory infection, and dying of any cause.

In the COVIDIOL trial in Cordoba, Spain, the above described pilot study among 76 participants [18] is followed by a trial involving 1008 patients aged 18–90 years diagnosed with COVID-19 and radiological image compatible with inflammatory pleuropulmonary exudate [24]. The intervention group receives the best available treatment plus oral calcefediol (0.532 mg = 21,280 IU on day 1, 0.266 mg = 10.640 IU on days 3, 7, 14, 21 and 28), while the control group receives the best available treatment only. The primary endpoints are ICU admission and deaths within 28 days. The secondary endpoints include, among others, time from onset of symptoms to discharge of patients in conventional hospitalization, time until admission to ICU with mechanical ventilation, and time until mechanical ventilation is removed.

In the CoVitTrial, a multicenter randomized trial conducted in France, 260 high-risk patients aged 65 year or older diagnosed with COVID-19 infections within the preceding 3 days and seen in hospitalization or consultation or in nursing home are recruited [25]. The trial compares the offer of a single high oral dose of vitamin D3 (400,000 IU) with the offer of a single low oral dose of vitamin D3 (50,000 IU). The primary endpoint is death from any cause during the 14 days following the inclusion and intervention. The secondary endpoints include, among others, death from any cause during 28 days and clinical evolution during 14 days and 28 days based on the change of the World Health Organization (WHO) Ordinal Scale for Clinical Improvement for COVID-19.

In a pragmatic, cluster randomized, double-blinded trial in the United States (The vitamin D for COVID-19 (VIVID) trial, *n* = 2700 in total nationwide), 1500 newly diagnosed individuals with COVID-19, together with up to one close household contact each (~1200 contacts), are recruited nation-wide via social media; community advocacy groups and equity initiatives; and flyers and electronic communications distributed in healthcare centers, low income residential housing organizations, COVID-19 testing centers, and other avenues. Participants are randomized to either vitamin D3 (9600 IU/day loading dose on days 1 and 2, then 3200 IU/day) or placebo in a 1:1 ratio and a household cluster design [26]. The study duration is 4 weeks. The primary outcome for newly diagnosed individuals is the occurrence of hospitalization and/or mortality. Key secondary outcomes include symptom severity scores among cases and changes in the infection (seroconversion) status for their close household contacts. Changes in vitamin D 25(OH)D levels will be assessed and their relation to study outcomes will be explored.

## 5. Public Health and Clinical Implications

Vitamin D insufficiency and deficiency are widespread in many countries, in particular among the elderly, calling for public health action even before the COVID-19 pandemic [27]. Randomized controlled trials (RCTs) have shown efficacy and safety of vitamin D supplementation in preventing various adverse health outcomes, such as hip fractures, acute respiratory infections, or deaths from cancer [28,29,30]. Widespread vitamin D supplementation, at least for the elderly and the high risk groups, thus seems to be prudent even in the absence of the COVID-19 pandemic and is recommended or practiced to some extent in a few countries [31]. Nevertheless, vitamin D levels have remained inadequate in most countries [27], with prevalence of vitamin D deficiency remaining highest among nursing home residents [32], the group at highest risk for COVID-19 infection and death. The ongoing COVID-19 pandemic, globally accounting for more than 10 million new cases and 200,000 deaths per month in the second half of 2020, calls for immediate efforts to enhance vitamin D status of populations at risk and of those infected with COVID-19 even before results of the ongoing large trials become available, which will not be before spring to summer of 2021. Besides the recent epidemiological evidence outlined above, a major protective impact of vitamin D supplementation on risk and course of COVID 19 infections is strongly supported by long known and well established molecular mechanisms of vitamin D, such as its immunomodulatory effects, as outlined in detail elsewhere [2,33,34,35,36,37]. Vitamin D supplementation could thus be a most cost-effective, readily available tool that could potentially prevent millions of COVID-19 infections and tens if not hundreds of thousands COVID-19 deaths, and at the same time, prevent overstretching of health care systems, beyond its established beneficial effects on other health outcomes.

Obviously, vitamin D supplementation should complement, not replace established and other efforts to cope with the COVID-19 pandemic, such as social distancing, wearing of masks, and hygiene measures with which it shares protective effects not only against COVID-19 infections, but also other infections, such as other acute respiratory infections including influenza. Although there is hope that widespread vaccination will finally end or at least widely control the current COVID-19 pandemic, its long-term safety and effectiveness are yet to be demonstrated. In the meantime, but also in the long run, vitamin D supplementation, for which safety and effectiveness with respect to acute respiratory infections has long been established, and which is a very low-cost measure, should be widely applied. Even a minor effect on protection from infection that might turn the COVID-19 effective reproduction number from slightly above one (as estimated for many countries shortly before or during lockdown measures of varying intensity during most of the second half of 2020) to slightly below one could make the difference between further exponential growth or regression of the pandemic. In the absence of specific contraindications, supplementation with safe, but sufficient doses (e.g., ranging from 800 to 4000 IU/day for older adults depending on individual factors, such as age and sex [38,39], body mass index, or comorbidity) should thus be strongly promoted for the population at large and the high-risk population in particular, not only to those with already manifest COVID-19 infection. Despite remaining uncertainties with respect to optimal dosing, evidence from vitamin D trials with other endpoints suggests supplementation should preferably be done on a regular basis rather than by occasional high-dose bolus therapy. For patients with manifest COVID-19 infection, initiation of high-dose supplementation as early as possible after diagnosis should be strongly considered whenever there are no specific contraindications against such treatment. At the very least, such strategies would help to reduce the burden of established adverse consequences of widespread vitamin D insufficiency and deficiency, which would be a great achievement by itself. In the best case, they might add to this the even greater achievement of curbing the ongoing COVID-19 pandemic with all its adverse consequences even prior to and beyond widespread availability of vaccination. Immediate action is warranted.

## Data Availability

Not applicable.
